# The role of sample-specific stressors, social identification, stress mindset and resilience for the well-being of descendants of mentally ill parents: a quasi-experimental cross-sectional study

**DOI:** 10.1186/s12888-026-08361-x

**Published:** 2026-10-02

**Authors:** Isabel Grünenwald-Mattern, Luise Beyer, Antonia J. Kaluza, Rolf van Dick

**Affiliations:** 1 Department of Psychology, Goethe University, Theodor-W.-Adorno-Platz 6, 60323 Frankfurt, Germany; 2 University of Tübingen, Tübingen, Germany; 3 Lingnan University, Hong Kong, China

**Keywords:** Descendants of mentally ill parents, Social identification, Stress mindset, Resilience, Well-being, Preventive health research

## Abstract

**Background:**

Descendants of mentally ill parents (DMIP) make up about 22% of the population and are 2–13 times more likely to experience mental problems themselves. Preventive health interventions are needed, which should foster protective factors. Unfortunately, little is known about which variables can serve as protective factors for this at-risk group.

**Methods:**

In this quasi-experimental cross-sectional study, we compared DMIP (*n* = 332) with a control group (*n* = 90) with mentally healthy parents. We predicted that (1) DMIP report a lower well-being than the control group and that DMIP who experienced more sample-specific stressors report lower well-being, (2) that family identification relates to better well-being but that this relationship is moderated by ambivalent identification and disidentification and (3) that the relationship between sample-specific stressors and well-being is moderated by resilience and stress mindset.

**Results:**

Analyses show that compared to a control group, DMIP rather have a negative stress-mindset, identify less with their family, show higher disidentification and ambivalent identification with their families and report poorer well-being for most measures. DMIP who experience more sample-specific stressors report lower well-being. Identification with the family positively relates to DMIP’s well-being. This relationship was especially strong when ambivalent identification was low (for DMIP but not for the control group). The relationship between the sample-specific stressors and well-being is not moderated by resilience or the stress mindset.

**Conclusions:**

For preventive health programs, we propose to assess specific stressors and tailor interventions accordingly; to integrate modules which foster identification with the family and reduce ambivalent identification and to test if a stress mindset training benefits DMIP.

**Clinical trial number:**

Not applicable.

## Background

The life of the parents is the book in which the children read.

(Augustinus Aurelius)

A child’s home statistically influences the development of a human being massively, but no child can influence which book they get to read from their parents. For instance, studies have shown, that families with a migration background are more at risk of poverty [1]. Also, the level of a child’s attained education is heavily influenced by the parents’ socioeconomic status [2]. Those two examples illustrate, that parents play a central role for the development of their children. One aspect of how children are influenced by their parents, which is not sufficiently studied yet, is the parent’s mental health status [3].

The number of affected children with parents who have a mental illness is alarming: Approximately one third of all adults develop a mental illness over their lifespan [4]. Out of these, 60–70% are also parents [5]. Conservative estimations assume, that there are approximately three million children with at least one mentally ill parent in Germany, which accords to about 22% of the population. A comparable number is known from Switzerland and can be estimated in other countries in Europe and the USA [6]. In addition, there might be high unreported numbers [7]. Nevertheless, in the scientific field, people with a mental illness are rarely looked at in their parental role [7].

Children of mentally ill parents have a high strain compared to other children and they are two to 13 times more likely to develop a mental illness themselves [8–12]. Furthermore, maternal mental illness has been shown to have an influence on the child’s developmental vulnerability [13]. Therefore, and because of the high prevalence as shown above, it is very important to research how exactly the well-being of those children is influenced by their parents’ mental disease and which variables could serve as protective factors for the children’s well-being. Those children have little control about changing the mental illness or behavior of the parent, thus it is of high importance to look at what they do have control over, in order to protect their own mental health and development. When talking about protective factors, one important factor is a person’s resilience, i.e. the ability of an individual to remain healthy in the face of psychological, biological, or psychosocial developmental risks [14]. In this study, we look at how resilience moderates the relationship between specific stressors, which children of mentally ill parents experience, and their well-being. Furthermore, we will study potential protective factors which are more susceptible. Two such factors, which have shown to be influential for the well-being of other populations with chronic stressors (e.g., chronic pain patients), are the stress mindset, i.e. the belief that stress itself has enhancing or debilitating consequences [15], and social identification, i.e. a person’s relationship to a social group and the positive emotional evaluation of this relationship with a person or a group [16, 17]. To the best of our knowledge, neither the influence of the stress mindset, nor of social identification have been studied in the context of descendants of mentally ill parents yet. We aim to identify, if those two factors play a role for the well-being of descendants of mentally ill parents, in order to detect potential susceptible protective factors for this burdened target group and in order to improve preventive health programs. We therefore assessed data from a group of descendants of mentally ill parents and from a control group with mentally healthy parents, compared them regarding those variables of interest and checked how those variables relate to each other. In doing so, we created a questionnaire about the sample-specific stressors, derived from literature based, part-structured interviews with two descendants of mentally ill parents.

## Theory and hypothesis development

**Fig. 1:**
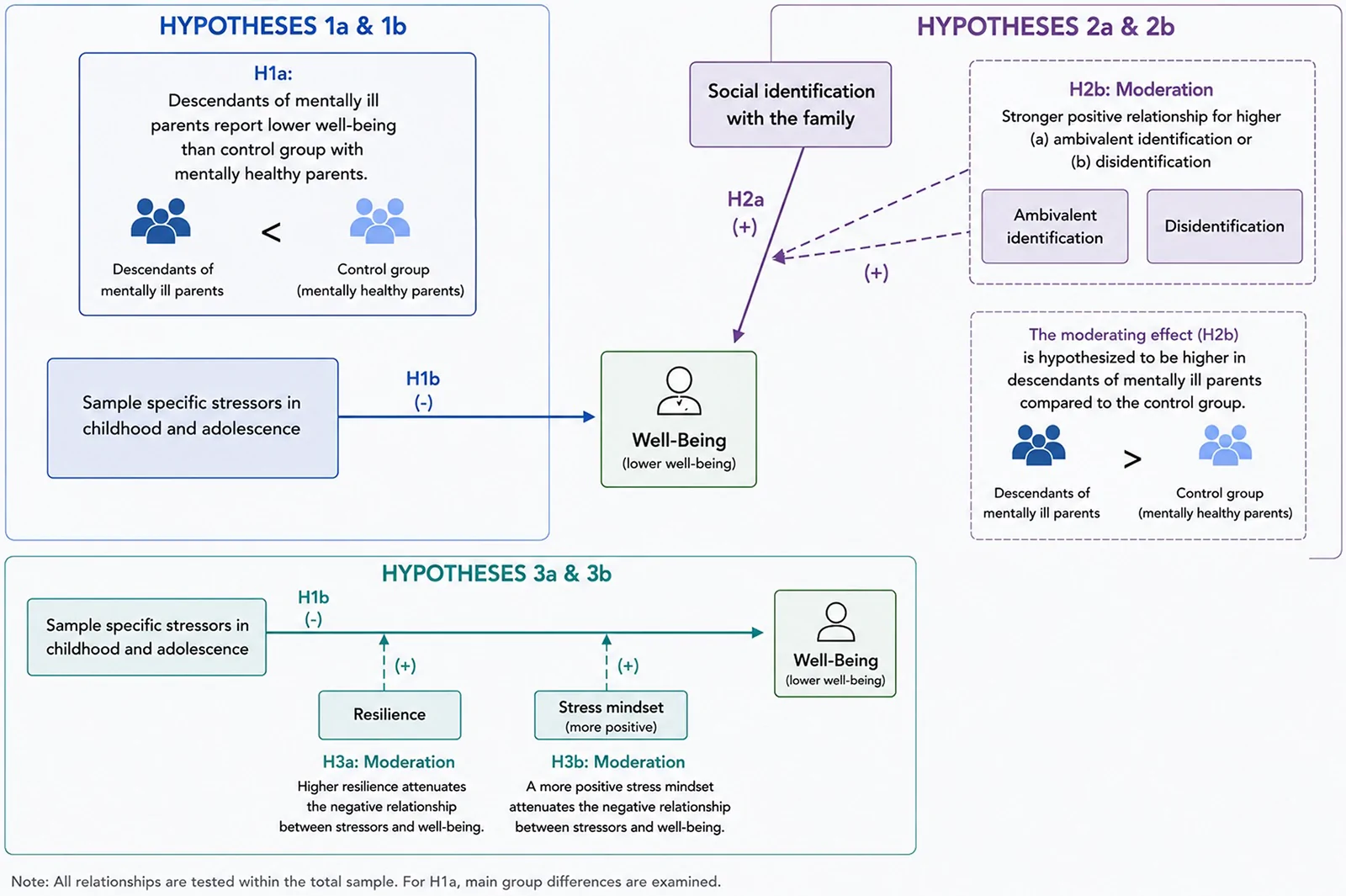
Conceptual model underlying the study hypotheses

As our hypotheses extend into various research areas and are therefore quite complex, they are summarized beforehand in Fig. 1, to assist reading and understanding of the following section.

### Stressor-intensity and well-being

What does it actually mean for a child to have mentally ill parents? The stress factors that affect the child should not be underestimated. Stressors are understood to be threatening stimuli that activate the body’s stress response [18]. In the context of mentally ill parents, special, sample-specific stressors affect the children. These numerous stressors specific for children of mentally ill parents represent risk factors for child development and are differentiated into (1) factors on the part of the parents and the family, (2) factors on the part of the children and (3) general psychosocial stress factors (see Table 1) [19]. An example of this is parentification, a role reversion in which the children adopt the parental role [20] and thus have to put their own needs on hold and take on overburdening responsibilities. However, rejecting responsibility creates a conflict of loyalties for the child. An equally widespread stressor is the taboo of parental illness: In many families, the illness of the parent is kept secret. In the study by Sollberger [21], 62% of the offspring of mentally ill parents stated that they did not talk about their parents’ illness outside the family, and 53% did not even talk about it within the family. This has a number of consequences: Firstly, children perceive changes that are not explained by the parents, which leads to irritation. Secondly, the lack of information inhibits an appropriate mobilization of resources. In addition, the silence imposed on the descendants creates enormous pressure, as they are torn between feelings of loyalty and shame and at the same time the need to confide [22]. The result of this inner conflict is often the feeling of being left alone. Since those children can hardly confide in anyone about their parents’ illness and the associated stress factors, the internal and external taboo within the family is closely linked to a lack of social support. This is very important as social support is a protective factor for well-being [22]. For example, Catherine Haslam and her colleagues [23] have found that group membership promotes social support and identification and thus strengthens cognitive health.

All this suggests that children with mentally ill parents suffer from sample-specific stressors in comparison to children of healthy parents. Thus, it is not far-fetched to consider that the well-being of affected children is also poor. Well-being is understood as “optimal psychological functioning and experience” [24, p. 142), which includes all three aspects of well-being: physical, psychological and social [25]. For example, mothers of autistic children have been shown to have more stressors and a higher risk of poor psychological well-being [26]. Since overwhelming stressors are by definition considered a threat to well-being [27], the well-being of descendants of mentally ill parents could therefore be severely compromised. As not all descendants of mentally ill parents are exposed to all potential sample-specific stressors mentioned above, we propose, that the more sample-specific stressors are experienced by those descendants, the lower their well-being is. Therefore, we predict:

#### Hypothesis **1a (H1a)**

Descendants of mentally ill parents report a lower well-being compared to a control group with mentally healthy parents.^1^

#### Hypothesis 1b (H1b)

Descendants of mentally ill parents who experienced more sample specific stressors in their childhood and adolescence report lower well-being.

**Table 1:** Overview of the different sample-specific stressors (Classification according to Wiegend-Grefe et al. (2011); own depiction)

Factors on the parent`s and family`s part	Factors on the children`s part	General psychosocial stressors
**Disease-related variables (Wiegand-Grefe et al., 2011)** • The severity of the disease • The chronicity • The diagnosis and specific stressors associated with it (e.g. Personality disorders are considered to be particularly risky for the children of those affected)	**Personality traits and soft skills (Wiegand-Grefe et al., 2011):** • Diminished intellectual and social skills • Poor communication skills • Little ability to take responsibility • Low self-esteem	**Low socio-economic status (Wiegand-Grefe et al., 2011)** • Low income • Restricted housing conditions
**Lower parenting skills (Wiegand-Grefe et al.**,** 2011)**: • Conflictual parental relationship • Low emotional availability • Psychological instability, which can lead to attachment and relationship problems • Parentification (Lenz, 2014) • Abuse • Neglect • Frequent, prolonged and early separation experiences • Care deficit of the children and a lack of parental attention	**Subjective emotional state of the children** • The concern of becoming ill themselves, anger and disappointment (Plass & Wiegand-Grefe, 2012) • Fear of loss, feelings of guilt (Remschmidt & Mattejat, 2008), • Grief and hopelessness (Lenz & Wiegand-Grefe, 2017). • The interconnectedness of these feelings also leads to increased irritability in the offspring (Lenz, 2014) • Being torn between loyalty and shame and at the same time the need to confide (Lenz, 2014) • Feeling of being left alone (Lenz, 2014)	**Structural ** **(Lenz & Wiegand-Grefe**,** 2017)**: • Breakdown of familiar structures and structures and routines • Lack of information about the disease • Lack of awareness about the disease. • Young age at first manifestation of the disease • Interactions between the factors that increase the stress beyond an additive effect
**Inadequacy of the family’s coping with the disease (Wiegand-Grefe et al.**,** 2011)**: • Tabooing of the illness • Unstable living conditions • Lack of communication • Lack of cohesion in the family		**Extrafamilial ** **(Wiegand-Grefe et al.**,** 2011)** • Lack of social support • Family isolation • Low emotional availability of caregivers outside the family

### The role of social identification

So far, we have established why descendants of mentally ill parents could have a lower well-being, due to their sample-specific stressors they are opposed to. In the following, we want to focus on factors which could possibly ameliorate the well-being of those descendants, starting with social identification. Social identification is defined as “the positive emotional valuation of the relationship between self and ingroup” [16, p. 599), where “ingroup” means a social group to which a person belongs. Social identification is an important basis for social support, which buffers stress [28], protects mental health [29], and increases well-being [30]. Furthermore, a high level of social identification is proven to have a beneficial outcome on psychological and physical health (e.g., [31, 32]). Van Dick and colleagues [33] could recently show that identification with more social groups, including the family, reduces stress and symptoms of ill-health. Therefore, we predict:

#### Hypothesis 2a (H2a)

Social identification with the family relates to better well-being in descendants of mentally ill parents.

However, if the group one identifies with is also a source of strain – as families with a mentally ill parent often are (e.g., [3]) – it might be helpful for one’s well-being to not fully identify with this group, but rather to disidentify with certain problematic aspects and/or have an ambivalent identification. The concept of ambivalent identification and disidentification is derived from the organizational psychological field. In this context, Kreiner and Ashforth [34] distinguished four dimensions of social identification: Identification, disidentification, ambivalent identification and neutral identification. Disidentification means, that an individual defines him or herself as not having the same attributes or principles that he or she believes define the social group [35, 36]. Ambivalent identification is defined as the simultaneous appearance of identification and disidentification with a group or aspects of it [34, 36, 37]. In this case, a person is pulled towards identification with a group in some dimensions but pulled towards disidentification on other dimensions [34].

Despite the positive effects of social identification as mentioned above, which are often referred to as “the social cure” (e.g., [31]), group identification can also decrease performance and well-being, which has been called “a social curse” [38–40]. The “social curse”, for example, manifests itself in that highly identified group members are more strongly affected by threats to their group, such as when an outgroup has a higher status because this group membership plays a more important role in their self-definition [41]. This might relate to descendants of mentally ill parents, as families with a mentally ill parent often do have a lower socio-economic status compared to families with mentally healthy parents [19]. Thus, it might not be as good for those descendants to identify very highly with their family, as they could suffer from integrating the lower socio-economic status into their self-definition. Furthermore, the “social curse” shows its effect in a way, that highly identified group members are more likely to show stress reactions when they observe another group member to be threatened [42]. This might also relate to descendants of mentally ill parents, as they notice their parents – as part of their social group they identify with – to be threatened by the mental illness and its consequences. Moreover, highly identified group members are less likely to share their problems with group members, as they want to protect them. This decreased tendency to share problems may result in a lack of receiving support from the group members [43]. This also applies to descendants of mentally ill parents: Talking about the parental illness and thus also about the strains that come with it for the child, is often tabooed even within the family (see 21). Furthermore, children of mentally ill parents often show signs of parentification, a role reversion in which the children adopt the parental role [20] and thus have to put their own needs on hold and take on overburdening responsibilities. Therefore, it might be important for the well-being of those descendants, to also show some extent of ambivalent or disidentification with their family, thus they could more easily talk about their strains, as they less feel like they need to protect their parents.

Another argument why a certain amount of ambivalent identification and / or disidentification might be helpful for descendants of mentally ill parents is the so-called norm enactment hypothesis [39]. According to this hypothesis, group members which highly identify with the group aim to enact a group’s values and norms. Thus, they perceive such norms as more prescriptive of appropriate cognition, affect and behaviors. As the cognition, affect and behaviors of people with a mental health condition are often distorted (e.g., [44]), it might be helpful for the well-being of their descendants to not fully identify with the affected parent or their family, because if they highly identify with them, they might perceive those distorted cognitions, affects and behaviors as appropriate and enact on them, which might lead to becoming mentally ill themselves or at least negatively impact their own well-being. Building on the argumentation described above we predict:

#### Hypothesis 2b (H2b)

The relationship between family identification and well-being as predicted in Hypothesis 2a is moderated by (a) ambivalent identification and (b) disidentification, in terms of a stronger relation for participants with higher ambivalent identification or disidentification. We hypothesize that this moderating effect is higher for descendants of mentally ill parents than for the control group with mentally healthy parents.

### Resilience and stress mindset

The stressors that children suffer from are often hardly avoidable. Therefore, it is important to analyze factors that could be protective for children’s well-being.

A classic protective factor related to stress is resilience. Resilience is the ability of an individual to develop healthily in the face of psychological, biological, or psychosocial developmental risks [14]. This occurs with the use of resources [45]. The ability to develop resilience is situation-specific and changes over the lifespan.

In this regard, research has shown that more resilient children developed psychologically healthy despite adverse life conditions, while less resilient children developed mental health issues under the same severe conditions [46]. Therefore, we assume that resilience also has a protective and beneficial effect on the development of children with mentally ill parents.

In addition, we investigate the role of another protective factor, which is easier to influence than resilience: The stress mindset, which is conceptualized by the researchers as “the extent to which one holds the belief that stress has enhancing consequences for various stress-related outcomes such as performance and productivity, health and wellbeing, and learning and growth (referred to as a “stress-is-enhancing mindset” (SIE)) or holds the belief that stress has debilitating consequences for those outcomes (referred to as a “stress-is-debilitating mindset” (SID))” [15, p. 716). The stress mindset is considered a significant factor in stress research, as it predicts health, performance and well-being over and above the usual stress-influencing factors such as stress intensity, subjective perception and evaluation [15]. While a stress-is-enhancing mindset correlates with, among other things, fewer symptoms of anxiety and depression as well as more energy and life satisfaction [15], a stress-is-debilitating mindset is associated with less cognitive flexibility and adaptability [47]. Since a stress-is-enhancing mindset increases life satisfaction and also has a positive impact on well-being [15, 17, 48], we hypothesize a moderating effect of the stress mindset on the well-being of descendants of mentally ill parents. In summary, we suggest a protective effect of resilience and of a stress-is-enhancing mindset on the well-being of descendants of mentally ill parents. Therefore, we predict:

#### Hypothesis 3a (H3a)

The assumed relationship from Hypothesis 1b that descendants of mentally ill parents who experienced more sample specific stressors in their childhood and adolescence report lower well-being is moderated by resilience, in terms of a higher resilience attenuating the negative effect from the sample specific stressors on well-being.

#### Hypothesis 3b (H3b)

The assumed relationship from Hypothesis 1b that descendants of mentally ill parents who experienced more sample specific stressors in their childhood and adolescence report lower well-being is moderated by the stress mindset, in terms of a more positive stress mindset attenuating the negative effect from the sample specific stressors on well-being.

## Methods

The aim of the study was to detect protective factors for the well-being of descendants of mentally ill parents, to improve preventive health programs.

### Participants and procedure

We received ethical approval by our institutional ethics committee to conduct this study. A quasi-experimental design was used, as descendants of mentally ill parents and descendants of mentally healthy parents are already existing and naturally occurring groups. As this study serves the purpose of providing a first overview about whether the variables of interest play a role for the well-being of descendants of mentally ill parents, we used a cross-sectional design.^2^ After being informed about the content and procedure of the study by a text on the first page of the questionnaire, participants gave their informed consent to contribute to the study and answered the questionnaire. Participants received no payments or other compensation for participating, but psychology students that participated in the control group could receive university credit points. Participants were recruited in the time of April till November 2022. We recruited both groups via social media platforms – the descendants of mentally ill parents were also recruited via special self-support groups on social media. The questionnaire for descendants of mentally ill parents was answered by a total of 371 people, the questionnaire for the control group was answered by a total of 123 people. In the control group, participants were excluded if they indicated to have a parent with a mental illness (this applied to 25 participants). In both groups, participants were excluded if they were under the age of 18, failed to answer at least two of the three attention check items or completed the questionnaire in less than 50% of the average completion time. For this criterion, we choose the median instead of the mean, since respondents have the option to pause their participation and continue it later. This sometimes results in long processing times, which would bias the mean. Using box plot analysis, only one outlier (in the control group) could be identified on the variable neutral identification. As this variable is not used in the hypotheses, we included this outlier in the analysis. After the application of the described exclusion criteria, our final sample consists of *n* = 332 descendants of mentally ill parents and *n* = 90 participants in the control group.

**Fig. 2:**
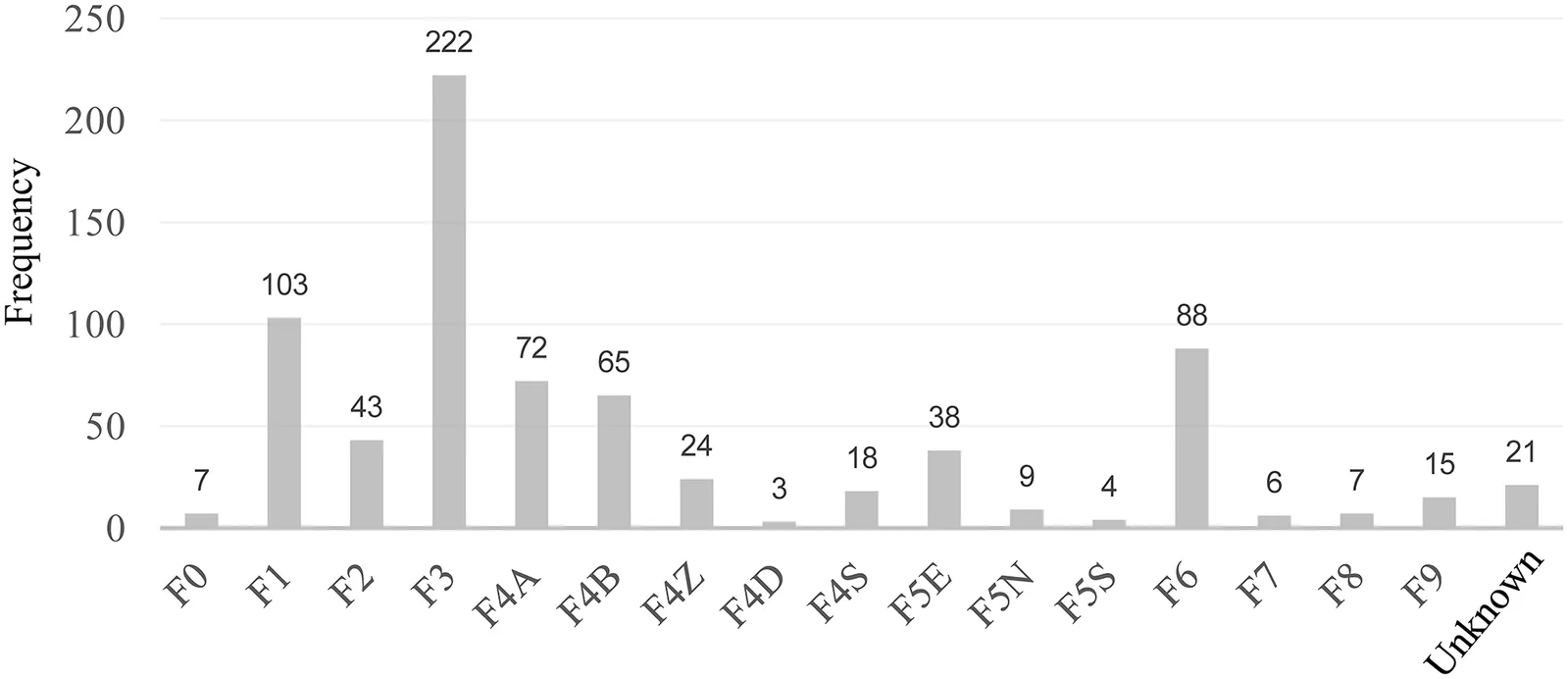
Distribution of parental illnesses (according to ICD-10 ([44]). Note. F0 = Organic, including symptomatic mental disorders; F1 = Mental and behavioral disorders due to psychoactive substance abuse; F2 = Schizophrenia, schizotypical and delusional disorders; F3 = Mood (affective) disorders; F4A = Neurotic disorders; F4B = Stress-related disorders; F4Z = Obsessive-compulsive disorders; F4D = Dissociative disorders; F4S = Somatoform disorders; F5E = Eating disorders; F5N = Non-organic sleep disorders; F5S = Sexual dysfunctioning disorders; F6 = Personality disorders; F7 = Mental retardation; F8 = Disorders of psychological development; F9 = Behavioral and emotional disorders with onset usually occurring in childhood and adolescence

In the group of descendants of mentally ill parents, the mean age is *M* = 32.74 (*SD* = 9.50), ranging between 18 and 64. In this group, 84% women, 14.8% men and 1.2% diverse people participated. The three most frequent highest educational achievements in this group were (1) a master degree (31.9%) (2), a bachelor degree (29.2%) and (3) high school leaving certificate (17.2%). The distribution of the parental diagnosis as indicated by the participants is depicted in Fig. 2.

In the control group, the mean age is *M* = 28.59 (*SD* = 9.58), ranging between 18 and 59. In this group, 84.4% women and 15.6% men participated. The three most frequent highest educational achievements in this group are (1) high school leaving certificate (36.7%) (2), a bachelor degree (20%) and (3) a master degree (14.4%).

### Measures

#### Subjective psychological well-being

To measure participants’ general well-being, we used the five-item Well-Being Index (WHO-5) based on Brähler and colleagues [49]. The participants rated items such as “Over the past 2 weeks I have felt cheerful and in good spirits” and “Over the past 2 weeks I have felt calm and relaxed” on a 6-point scale from *1 = At no time* to *6 = All of the time*. A sum score is calculated, which can range between 5 and 30, with higher values indicating a better well-being. The scale showed a reliability of Cronbach’s *α* = 0.84 in our sample.

We also used the German version of the General Health Questionnaire (GHQ-12) [50] with one additional item (“In the last two weeks I felt mistrustful”). The GHQ-12 has a 4-point response scale (corresponding to symptom present: *“not at all”*,* “same as usual”*,* “rather more than usual”*,* or “much more than usual”*). It can be scored in a bimodal fashion (0-0-1-1). Alternatively, it can be scored by using a 4-point response format (Likert-Scale: 0-1-2-3) [51]. We used the Likert-Scale approach, resulting in a sum score ranging from 0 to 39, with higher values indicating better well-being. Sample items are “In the last two weeks, I felt constantly under strain” or “In the last two weeks, I could enjoy day-to-day activities”. The scale showed a reliability of Cronbach’s *α* = 0.87 in our sample.

#### Social identification

To measure identification, disidentification and ambivalent identification, we used the scales of Kreiner and colleagues [34]. As their items were formulated to measure organizational identification, we adapted the items to the familial context. Each of the three scales was assessed by three items, which were rated on a 6-point scale from *1 = Strongly disagree* to *6 = Strongly agree*. Sample items are “When I talk about my family, I usually say ‘we’ and not ‘them’” (identification), “I want others to know, that I do not always agree with what my family does” (disidentification), “I have mixed feelings about belonging to my family” (ambivalent identification). A mean for each scale is calculated, which can range from 1 to 6, with higher values indicating a higher amount of identification / disidentification / ambivalent identification. The scale Identification showed a reliability of Cronbach’s *α* = 0.84 in our sample, Disidentification of *α* = 0.76, Ambivalent Identification of *α* = 0.89.

#### Resilience

We measured this construct using the German version of the six-item Brief Resilience Scale [52]. On a 5-point scale from *1 = Strongly disagree* to *5 = Strongly agree*, participants rated items like “I tend to bounce back quickly after hard times” or “I tend to take a long time to get over set-backs in my life”. A mean is calculated, which can range between 1 and 5, with higher values indicating a higher level of resilience. The scale showed a reliability of Cronbach’s *α* = 0.85 in our sample.

#### Stress mindset

We measured this construct using the eight-item Stress Mindset Measure (SMM) developed by Crum and colleagues [15]. On a 5-point scale from *1 = Strongly disagree* to *5 = Strongly agree*, participants rated items like “The effects of stress are positive and should be utilized” or “The effects of stress are negative and should be avoided”. A mean is calculated, which can range between 1 and 5. Higher values on this scale indicate an SIE mindset. The scale showed a reliability of Cronbach’s *α* = 0.85 in our sample.

#### Sample-specific stressors

To our best knowledge, there is no specific, already validated questionnaire or tool to assess the sample-specific stressors a descendant of a mentally ill parent experienced. Therefore, we developed a questionnaire for this purpose and for our investigation. Two adult descendants (both aged in their twenties) of a mentally ill parent (one with an alcoholic father and one with a father with a bipolar disorder) were interviewed about the stressors they experienced growing up with their mentally ill parent. We used a part-structured interview approach, with questions according to the typical stressors described by Wiegand-Grefe and colleagues [19]. A list of 26 sample-specific stressors was derived (see Appendix) and participants were asked to rate the extent to which they experienced this stressor in their childhood and adolescence on a 6-point scale from *1 = Not at all* to *6 = To a high amount*. A sum score is calculated, which can range between 26 and 156, higher values indicating a higher amount of experienced sample-specific stressors in the childhood and adolescence. The scale showed a reliability of Cronbach’s *α* = 0.91 in our sample.

As the sample-specific stressor scale was a self-developed scale, we computed an exploratory factor analysis (oblimin, as the factors are assumed to be correlated). The Kaiser-Meyer-Olkin Measure of Sampling Adequacy (KMO) is 0.91, indicating superb values [53]. The diagonal elements in the anti-image correlation matrix are above 0.50 or close to 0.50, which means that no values have to be excluded from the analysis [54]. The Scree Plot indicated a one factor solution (see Fig. 5 in the Appendix). Therefore, we decided for the one factor solution, i.e. a sum score above all sample-specific stressors. This approach is supported by the good reliability of Cronbach’s *α* = 0.91 of this scale.

### Data analysis

We conducted a priori power analyses, using G Power [55]. Following meta-analyses which explored the link between social identification and well-being (depression as a negative indicator of well-being; e.g., [32]), there is an overall small to moderate effect to expect. Concerning the relationship between stressors and well-being, a high effect can be expected, as shown by correlations between stress and mental well-being of *r* = − .67; *p* < .01 [56]. From these previous findings, we can assume an overall moderate effect size. Therefore, we computed the power analysis as following: Effect size f2 = 0.15; error probability = 0.05; Power = 0.95; Number of tested predictors = 3 (because of H2b, H3a, H3b). The power analysis showed that we needed 119 participants. 3

To test for common method bias, Harman’s single-factor test was performed by loading all self-reported variables into an unrotated factor analysis. The results revealed that the first factor accounted for only 18.47% of the total variance. Since this value is well below the threshold of 50%, common method bias is not a major concern in this study.

Data was analyzed using SPSS (version 29; [57]). For the moderation analyses, PROCESS v4.2 from Andrew F. Hayes was used [58]. The statistical analyses conducted were an ANOVA, regression and moderation analyses. We preregistered that we would compute all analyses with and without those participants, that indicated that their parents do have a mental health condition but no official diagnosis, and check if excluding them will change the results. However, this would have gone beyond the scope and lead to potentially excluding participants fitting in the group of descendants of mentally ill parents who simply might not know if their parents do have an official diagnosis or which one, which might be caused by the existing taboo to talk about the parental illness as described above. Having the high dark digit of this target group in mind, we thus decided to rather check if those participants were outliers. As they were not, we included them in the analyses.

## Results

### Descriptive statistics

Tables 2 and 3 present the means, standard deviations and correlations of the scale variables, as well as the reliabilities. Reliabilities ranged from *α* = 0.76 to *α* = 0.91, indicating acceptable or good consistency.

**Table 2:** Means (M), Standard Deviations (SD), correlations and reliabilities of the variables

	M	SD	1	2	3	4	5	6	7	8	9	10
1. DMIP vs. Control	-	-	-									
2. Age	31.86	9.66	− 0.176^**^	-								
3. Gender	-	-	− 0.017	− 0.044	-							
4. Stress Mindset	2.53	0.69	0.106^*^	− 0.066	0.036	(0.85)						
5. Identification	3.13	1.49	0.314^**^	− 0.139^**^	0.000	0.130^**^	(0.84)					
6. Ambivalent Identification	3.50	1.69	− 0.351^**^	0.134^**^	− 0.067	− 0.085	− 0.560^**^	(0.89)				
7. Disidentification	3.18	1.34	− 0.334^**^	0.058	− 0.034	− 0.093	− 0.483^**^	0.618^**^	(0.76)			
8. Resilience	2.95	0.87	0.192^**^	− 0.099^*^	0.081	0.428^**^	0.219^**^	− 0.242^**^	− 0.146^**^	(0.85)		
9. GHQ	19.24	6.64	0.075	0.013	0.057	0.238^**^	0.129^**^	− 0.129^**^	− 0.071	0.450^**^	(0.87)	
10. WHO	15.11	4.87	0.129^**^	− 0.065	0.031	0.292^**^	0.175^**^	− 0.174^**^	− 0.129^**^	0.437^**^	0.724^**^	(0.84)

Note. *N* = 422. DMIP = Descendants of mentally ill parents; DMIP vs. Control: 1 = DMIP, 2 = Control. Gender: 1 = female, 2 = male, 3 = diverse. GHQ = General Health Questionnaire (Well-Being Marker), WHO = Well-Being Index (Well-Being Marker). Cronbach’s Alpha is indicated in the brackets. *p < .05; **p < .01 (two-tailed). Higher values indicate higher peculiarity, for the stress mindset higher values indicate a stress-is-enhancing mindset. GHQ and WHO are sum scores, the rest are mean scores

**Table 3:** Means (M), Standard Deviations (SD), correlations and reliabilities of the variables only for DMIP

	M	SD	1	2	3	4	5	6	7	8	9	10
1. Age	32.74	9.50	-									
2. Gender	-	-	− 0.050	-								
3. Stressors	101.17	24.35	0.068	− 0.104	(0.91)							
4. Stress Mindset	2.49	0.69	− 0.054	0.040	− 0.123^*^	-						
5. Identification	2.89	1.45	− 0.099	0.039	− 0.351^**^	0.122^*^	-					
6. Ambivalent Identification	3.81	1.64	0.049	− 0.087	0.516^**^	− 0.068	− 0.506^**^	-				
7. Disidentification	3.42	1.33	− 0.007	− 0.022	0.501^**^	− 0.078	− 0.451^**^	0.542^**^	-			
8. Resilience	2.87	0.89	− 0.056	0.057	− 0.252^**^	0.446^**^	0.159^**^	− 0.178^**^	− 0.089	-		
9. GHQ	18.98	6.84	0.028	0.024	− 0.267^**^	0.246^**^	0.077	− 0.098	− 0.061	0.457^**^	-	
10. WHO	14.78	4.90	− 0.038	0.016	− 0.226^**^	0.307^**^	0.123^*^	− 0.131^*^	− 0.107	0.456^**^	0.741^**^	-

*Note. N* = 332 (only the descendants of mentally ill parents (DMIP)). Gender: 1 = female, 2 = male, 3 = diverse. GHQ = General Health Questionnaire (Well-Being Marker),

WHO = Well-Being Index (Well-Being Marker). *p < .05; **p < .01 (two-tailed). Higher values indicate higher peculiarity, for the stress mindset higher values indicate a

stress-is-enhancing mindset. Stressors, GHQ and WHO are sum scores, the rest are mean score

### Testing

We preregistered all analyses and conducted some exploratory analyses which were not preregistered. Those not preregistered ones are marked.

First, we tested, if there were differences between the two groups on the study variables^4^, including testing H1a, which assumes that descendants of mentally ill parents report a lower well-being compared to the control group with mentally healthy parents. An ANOVA shows that the descendants of mentally ill parents (*M* = 2.49, *SD* = 0.69) compared to control participants (*M* = 2.67, *SD* = 0.64) show a significant difference concerning their stress mindset, *F*(1, 420) = 4.79, *p* = .029, indicating that descendants of mentally ill parents rather have a stress-is-debilitating mindset than control participants. The ANOVA shows that the descendants of mentally ill parents (*M* = 2.89, *SD* = 1.45) compared to control participants (*M* = 4.03, *SD* = 1.28), show significantly less identification with their family, *F*(1, 420) = 45.88, *p* < .001. In addition, the descendants of mentally ill parents (*M* = 3.42, *SD* = 1.33) compared to control participants (*M* = 2.32, *SD* = 1.02) show significantly more disidentification with their family *F*(1, 420) = 52.67, *p* < .001. The descendants of mentally ill parents (*M* = 3.81, *SD* = 1.64) compared to control participants (*M* = 2.36, *SD* = 1.33) also show significantly more ambivalent identification with their family *F*(1, 420) = 59.06, *p* < .001. Concerning well-being, the descendants of mentally ill parents (*M* = 18.98, *SD* = 6.84) compared to control participants (*M* = 20.20, *SD* = 5.77) show no significant difference in the GHQ *F*(1, 420) = 2.41, *p* = .122. Using the WHO as a well-being indicator, the descendants of mentally ill parents (*M* = 14.78, *SD* = 4.90) compared to people without a parent with a mental health condition (*M* = 16.31, *SD* = 4.56) report significantly lower well-being *F*(1, 420) = 7.11, *p* = .008. The descendants of mentally ill parents (*M* = 2.87, *SD* = 0.89) compared to control participants (*M* = 3.27, *SD* = 0.75) show significantly less resilience *F*(1, 420) = 16.03, *p* < .001. Therefore, H1a is partially supported, depending on which well-being indicator is being used.

To test H1b, a regression analysis was computed (Table 4). We hypothesized, that descendants of mentally ill parents who experienced more sample specific stressors in their childhood and adolescence report lower well-being. The amount of experienced stressors in the childhood and adolescence could explain 7.1% of the variance of well-being being measured by the GHQ (*R*^*2*^ = 0.07, *F*(1, 330) = 25.40, *p* < .001). For the WHO as an indicator of well-being, 5.1% of the variance could be explained by the amount of experienced stressors in the childhood and adolescence (*R*^*2*^ = 0.05, *F*(1, 330) = 17.84, *p* < .001). Thus, H1b is supported by our results.

**Table 4:** Regression analysis to test H1b

	Well-being (GHQ)						
	*R*	*R* ^2^	ad. *R*^2^	b	SE	β	t
Intercept				26.58**	1.55		17.15
Stressors	0.27	0.07	0.07	− 0.08**	0.02	− 0.27	-5.04

	Well-being (WHO)						
	R	R^2^	ad. R^2^	b	SE	β	t
Intercept				19.40**	1.12		17.26
Stressors	0.23	0.05	0.05	− 0.05**	0.01	− 0.23	-4.22

*Note. N* = 332 (descendants of mentally ill parents). *p < .05; **p < .01 (two-tailed); ad. = adjusted, SE = Standard Error

To test Hypothesis 2a, a regression analysis was computed (Table 5). It was hypothesized that social identification with the family improves well-being in descendants of mentally ill parents. The identification with the family could explain 1% of the variance of well-being being measured by the GHQ (*R*^*2*^ = 0.01, *F*(1, 330) = 1.98, *p* = .16). The regression from the identification with the family to well-being (GHQ) is not significant, thus H2a is not supported by those results. For the WHO as an indicator of well-being, the regression from the identification with the family to well-being was significant, supporting H2a. For this model, identification with the family could explain 2% of the variance of well-being (*R*^*2*^ = 0.02, *F*(1, 330) = 5.06, *p* = .025). Therefore, H2a is partially supported, depending on which indicator of well-being is used.

**Table 5:** Regression analysis to test H2a

	Well Being (GHQ)						
	*R*	*R* ^2^	ad. *R*^2^	b	SE	β	t
Intercept				17.93**	0.83		21.51
Family Identification	0.08	0.01	0.00	0.36	0.26	0.08	1.41

	Well Being (WHO)						
	R	R^2^	ad. R^2^	b	SE	β	t
Intercept				13.59**	0.60		22.83
Family Identification	0.12	0.02	0.01	0.41*	0.18	0.12	2.25

*Note. N* = 332 (descendants of mentally ill parents). *p < .05; **p < .01 (two-tailed); ad. = adjusted, SE = Standard Error

To test Hypothesis 2b, moderation analyses were calculated. We hypothesized that the relationship from Hypothesis 2a is moderated by ambivalent identification and disidentification, in terms of well-being being better in descendants which score higher on ambivalent identification or disidentification. We compared this moderation model to a control group of people with parents without a mental health condition. We hypothesized that the moderating effect of ambivalent identification and disidentification is higher for descendants of mentally ill parents than for descendants of parents without a mental health condition. Therefore, we computed eight moderation analyses: Four for the descendants of mentally ill parents (varying using the WHO or the GHQ as the dependent variable (y) and ambivalent identification or disidentification as the moderator (M) and likewise four for the control group of people without a mentally ill parent. Only the four moderations for the group of descendants of mentally ill parents are depicted in detail here, the ones for the control group can be found in short in Tables 6 and 7, which summarize the results of the moderation analyses.

**Fig. 3:**
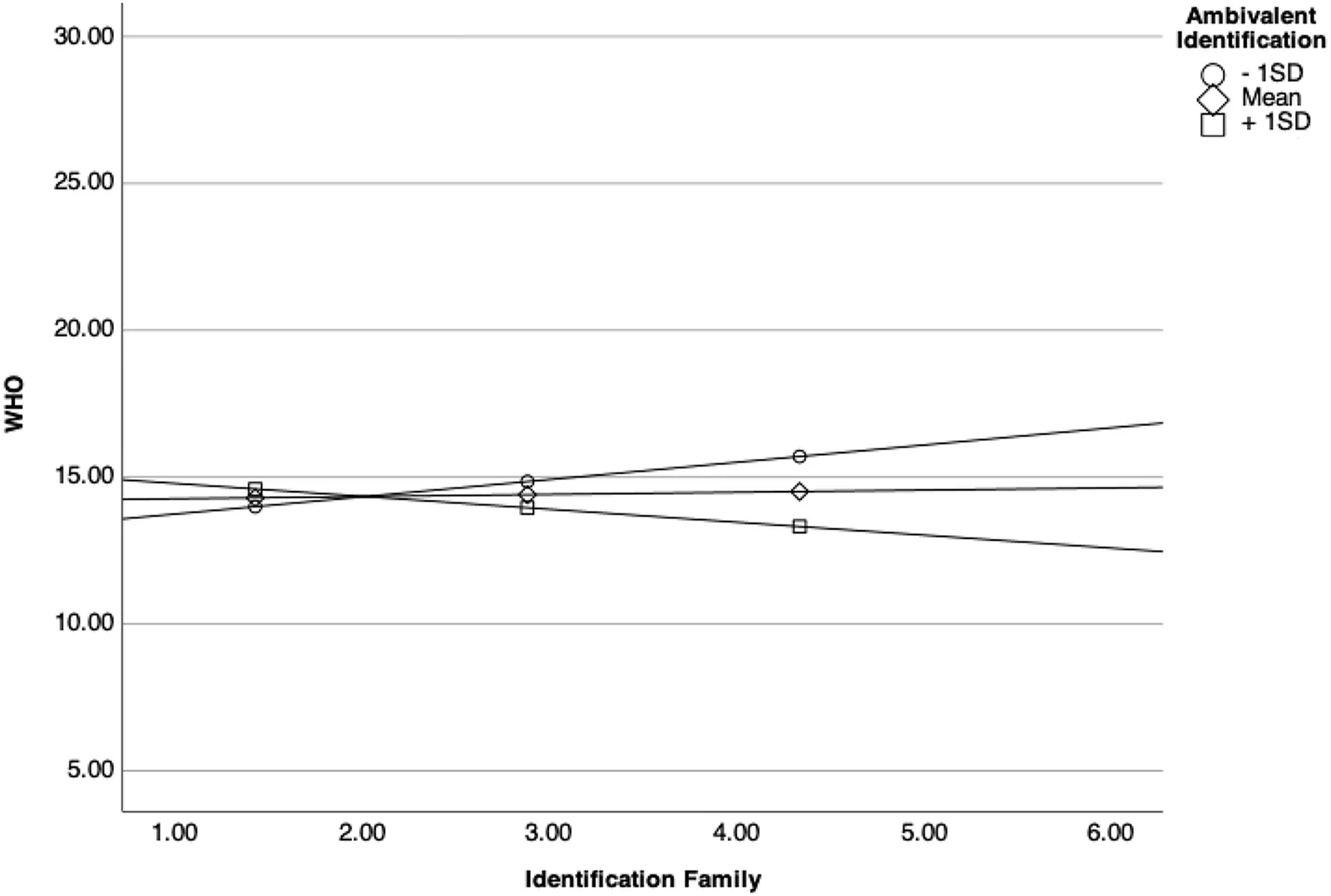
Simple slopes for H2b (y = WHO, M = Ambivalent Identification)

For the moderation analysis to test H2b using the WHO as dependent variable and ambivalent identification as a moderator, the results indicate that the relationship of identification with the family and well-being would be stronger if ambivalent identification is low. The results show that identification with the family (*b* = 1.27, *SE* = 0.43, *p* = .003) significantly positively relates to well-being, however, ambivalent identification with the family (*b* = 0.63, *SE* = 0.38, *p* = .100) does not. In addition, there is a significant interaction between identification with the family and ambivalent identification with the family (*b* = − 0.31, *SE* = 0.12, *p* = .007). Particularly, the analysis shows, that when ambivalent identification is low (-1SD), there is a significant positive relationship between identification with the family and well-being, *b* = 0.59, *SE* = 0.24, *p* = .017. But when ambivalent identification is high (+ 1SD), there is a non-significant negative relationship between identification with the family and well-being, *b* = − 0.44, *SE* = 0.33, *p* = .186. Figure 3 depicts the simple slopes for this analysis. The model explains 4.3% of variance (*R*^*2*^ = 0.04, *F(3*,*328)* = 4.91, *p* = .002).

**Fig. 4:**
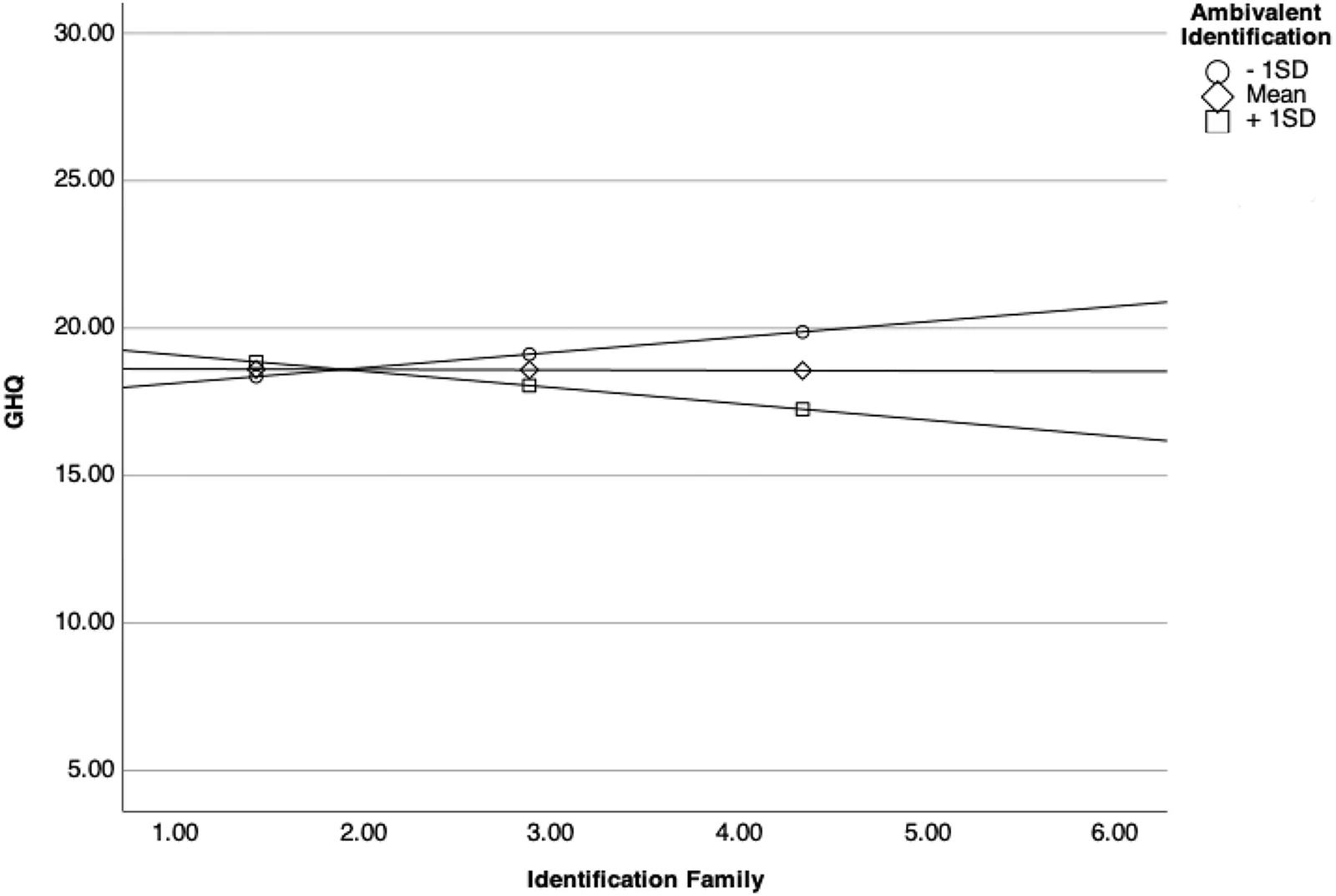
Simple slopes for H2b (y = GHQ, M = Ambivalent Identification)

For the moderation analysis to test H2b using the GHQ as a dependent variable and ambivalent identification as a moderator, the results indicate that the relationship of identification with the family and well-being would be stronger if ambivalent identification is low. The results show that identification with the family (*b* = 1.23, *SE* = 0.60, *p* = .042) significantly positively relates to well-being, however, ambivalent identification with the family (*b* = 0.62, *SE* = 0.54, *p* = .251) does not. In addition, there is a significant interaction between identification with the family and ambivalent identification with the family (*b* = − 0.33, *SE* = 0.16, *p* = .045). Particularly, the analysis show, that when ambivalent identification is low (-1SD), there is a non-significant positive relationship between identification with the family and well-being, *b* = 0.52, *SE* = 0.34, *p* = .130. But when ambivalent identification is high (+ 1SD), there is a non-significant negative relationship between identification with the family and well-being, *b* = − 0.55, *SE* = 0.47, *p* = .239. Figure 4 depicts the simple slopes for this analysis. The model explains 2.3% of variance (*R*^*2*^ = 0.02, *F(3*,*328)* = 2.54, *p* = .057).

**Table 6:** Moderation model testing H2b for ambivalent identification

DMIP	Well-Being (WHO) ***b***	Control Group	Well-Being (WHO) ***b***
Identification Family (IF)	1.27**	Identification Family (IF)	1.03
Ambivalent Identification (AI)	0.63	Ambivalent Identification (AI)	0.20
Interaction (IF x AI)	− 0.31**	Interaction (IF x AI)	− 0.13
*R* ^*2*^	0.04**	*R* ^*2*^	0.06

**DMIP**	**Well-Being (GHQ)** ***b***	**Control Group**	**Well-Being (GHQ)** ***b***
Identification Family (IF)	1.23*	Identification Family (IF)	1.48
Ambivalent Identification (AI)	0.62	Ambivalent Identification (AI)	0.25
Interaction (IF x AI)	− 0.33*	Interaction (IF x AI)	− 0.12
*R* ^*2*^	0.02	*R* ^*2*^	0.08

Note. *p < .05, **p < .01; DMIP = Descendants of mentally ill parents

For the moderation analysis to test H2b with using the WHO as a dependent variable and disidentification as a moderator, there could neither be found a significant relationship between identification with the family (*b* = 0.28, *SE* = 0.47, *p* = .549), nor disidentification (*b* = − 0.27, *SE* = 0.43, *p* = .533) and well-being. There was also no significant interaction between identification with the family and disidentification (*b* = 0.01, *SE* = 0.14, *p* = .936). The model explains 1.8% of variance (*R*^*2*^ = 0.02, *F(3*,*328)* = 2.05, *p* = .107). For the moderation analysis to test H2b using the GHQ as a dependent variable and disidentification as a moderator, there could also neither be found a significant relationship between identification with the family (*b* = 0.25, *SE* = 0.66, *p* = .705), nor disidentification (*b* = − 0.21, *SE* = 0.60, *p* = .733) and well-being. There was also no significant interaction between identification with the family and disidentification (*b* = 0.02, *SE* = 0.20, *p* = .941). The model explains 0.7% of variance (*R*^*2*^ = 0.01, *F(3*,*328)* = 0.75, *p* = .522).

**Table 7:** Moderation model testing H2b for disidentification

DMIP	Well-Being (WHO) ***b***	Control group	Well-Being (WHO) ***b***
Identification Family (IF)	0.28	Identification Family (IF)	0.88
Disidentification (DI)	− 0.27	Disidentification (DI)	0.20
Interaction (IF x DI)	0.01	Interaction (IF x DI)	0.00
*R* ^*2*^	0.02	*R* ^*2*^	0.06

**DMIP**	**Well-Being (GHQ)** ***b***	**Control group**	**Well-Being (GHQ)** ***b***
Identification Family (IF)	0.25	Identification Family (IF)	1.51
Disidentification (DI)	− 0.21	Disidentification (DI)	0.76
Interaction (IF x DI)	0.02	Interaction (IF x DI)	− 0.05
*R* ^*2*^	0.01	*R* ^*2*^	0.09*

Note. *p < .05, **p < .01. DMIP = Descendants of mentally ill parents

Taken together, those results do not support Hypothesis 2b. However, the results reveal a different interesting connection, namely that there is in fact an interaction effect between identification with the family and ambivalent identification, but in the opposite direction than we expected: It appears that rather than it being beneficial for one’s well-being if ambivalent identification with the family is high (as hypothesized), it is beneficial for one’s well-being if ambivalent identification is low, while identification is high. We could not find any moderating effect of ambivalent identification or disidentification on the relationship between family identification and well-being (regardless which well-being indicator was used) for the control group (see Tables 6 and 7). Therefore, this effect that it is especially beneficial for one’s well-being if family identification is high while ambivalent identification is low, could only be found for the descendants of mentally ill parents, not for the control group.

To test H3a and H3b, moderation analyses were conducted. Those are summarized in Table 8. It was hypothesized, that the assumed relationship from Hypothesis 1 is moderated by resilience (H3a) and by the stress mindset (H3b), in terms of a higher resilience and a more positive stress mindset attenuating the negative relationship between the sample specific stressors and well-being. For the moderation with resilience as a moderator (H3a), there was a significant relationship between the number of stressors in the childhood and adolescence (*b* = − 0.09, *SE* = 0.05, *p* = .049), but not for resilience (*b* = 1.61, *SE* = 1.57, *p* = .307) and well-being (measured by the GHQ). There was also no significant interaction between the number of stressors in the childhood and adolescence and resilience (*b* = 0.02, *SE* = 0.02, *p* = .292). The model explains 23.63% of variance (*R*^*2*^ = 0.24, *F(3*,*328)* = 33.84, *p* < .001). For the moderation with resilience as a moderator, using the WHO as an indicator of well-being, there was neither a significant relationship between the number of stressors in the childhood and adolescence (*b* = − 0.05, *SE* = 0.03, *p* = .157), nor for resilience (*b* = 1.54, *SE* = 1.14, *p* = .178) and well-being (measured by the WHO). There was also no significant interaction between the number of stressors in the childhood and adolescence and resilience (*b* = 0.01, *SE* = 0.01, *p* = .456). The model explains 22.23% of variance (*R*^*2*^ = 0.22, *F(3*,*328)* = 31,25, *p* < .001). Therefore, H3a is not supported. For the moderation with stress mindset as a moderator (H3b), there was no significant relationship between the number of stressors in the childhood and adolescence (*b* = − 0.05, *SE* = 0.05, *p* = .372), nor the stress mindset (*b* = 3.03, *SE* = 2.14, *p* = .159) and well-being (measured by the GHQ). There was also no significant interaction between the number of stressors in the childhood and adolescence and the stress mindset (*b* = − 0.01, *SE* = 0.02, *p* = .669). The model explains 11.82% of variance (*R*^*2*^ = 0.12, *F(3*,*328)* = 14.66, *p* < .001). For the moderation with stress mindset as a moderator and using the WHO as an indicator of well-being, there could also neither be found a significant relationship between the number of stressors in the childhood and adolescence (*b* = − 0.05, *SE* = 0.04, *p* = .208), nor the stress mindset (*b* = 1.67, *SE* = 1.53, *p* = .275) and well-being (measured by the WHO). There was also no significant interaction between the number of stressors in the childhood and adolescence and the stress mindset (*b* = 0.00, *SE* = 0.02, *p* = .821). The model explains 13.06% of variance (*R*^*2*^ = 0.13, *F(3*,*328)* = 16.42, *p* < .001). Thus, H3b was not supported.

**Table 8:** Moderation model testing H3a and b

	Well-Being (GHQ)***b***	Well-Being (WHO)***b***
Sample-specific stressors	− 0.09*	− 0.05
Resilience	1.61	1.54
Interaction	0.02	0.01
*R* ^*2*^	0.24**	0.22**

	**Well-Being (GHQ)** ***b***	**Well-Being (WHO)** ***b***
Sample-specific stressors	− 0.05	− 0.05
Stress Mindset	3.03	1.67
Interaction	− 0.01	0.00
*R* ^*2*^	0.12**	0.13**

Note. *p < .05, **p < .01

## Discussion

In our study, we were able to show that – as predicted – descendants of mentally ill parents reported poorer well-being compared to a control group with mentally healthy parents and that sample-specific stressors in particular were associated with poor well-being. Identification with the family positively related to higher well-being in descendants of mentally ill parents (using WHO as a well-being indicator) and this relationship was especially high when ambivalent identification was low. For stress mindset and resilience, we found no moderating effect. However, we found that people with mentally ill parents have a more negative stress mindset, thus perceive stress as something negative and harmful.

Our finding that in comparison to people without a parent with a mental health condition, descendants of mentally ill parents report lower well-being, is in accordance with previous literature. For example, Wiegand-Grefe and colleagues [59] show that the risk to develop psychopathological symptoms is three to seven times higher in children of mentally ill parents than in children of the general population. They also show that the quality of life is substantially decreased in children of mentally ill parents. Furthermore, we found that within the group of descendants of mentally ill parents, those who experienced more stressors relating to their parent’s mental health condition reported a worse well-being. This is in line with existing literature, e.g. according to Silverman [60], the degree of psychiatric disturbance in children of mentally ill parents depends on different factors, such as the degree of psychosocial stress experienced by the child, but also on the degree of the genetic loading, the intensity, frequency, duration and severity of the parental illness, the availability of an alternate healthy caretaker and the intensity and duration of exposure to the parents dysfunctional behavior.

Contrary to our assumptions, neither resilience nor stress-mindset moderated the relationship between sample-specific stressors and well-being. We did not find a study which tested this exact relationship, especially not with the number of sample-specific stressors of descendants of mentally ill parents as an independent variable. Nevertheless, our findings seem to contradict previous findings. Noor and Alwi [61] could find a moderating and a mediating role for resilience for the relationship between stressors and well-being in a sample of low socio-economic adolescents. They argue that resilience can be tested in a moderating or a mediating sense regarding the relationship between stressors and well-being. For example, Pretis and Dimova [62] describe that a parent’s mental illness always represents a stressor, but that the adverse effects associated with the parental disorder can be minimized or prevented by resilience factors. As explorative analyses reveal that the descendants of mentally ill parents report significantly lower resilience compared to people with mentally healthy parents and as the buffering effects of resilience are well-established (see above), this hypothesis should be retested by future research. Maybe the fact that we assessed sample-specific stressors as a retrospective self-assessment variable caused that we could not find a moderating effect of resilience on the relationship between sample-specific stressors and well-being. Possibly people who score higher on resilience have a positively distorted view on their stressors in their childhood and adolescence, which could have biased our results.

For the stress mindset as a moderator, our results of a non-existing moderation contradict previous findings in other samples, who are also exposed to stressful life events. For example, Jiang and colleagues [63] found that for migrant adolescents, stressful life events were positively associated with depression and negatively with life satisfaction, whereas those adolescents with a stress-is-enhancing mindset were less prone to depression when confronted with more stressful life events. Thus, in this sample, stress-mindset did serve as a protective factor for the migrant adolescent’s well-being. The positive outcomes of a stress-is-enhancing mindset for well-being are well established [15, 17, 48] and interestingly, exploratory analyses reveal that descendants of mentally ill parents rather report a stress-is-debilitating mindset compared to people with mentally healthy parents and thus suffer from a potential deficit. The stress-mindset of descendants of mentally ill parents has to our best knowledge never been contrasted with the stress-mindset of people with mentally healthy parents. Nevertheless, those findings seem to align with existing literature. Mattejat and Remschmidt [64] state that nearly all of the major sources of psychosocial stress which contribute to a child’s risk of mental illness are highly prevalent in families with a mentally ill parent. Ben-Avi and colleagues [65] assume that it is harder for people to develop a stress-is-enhancing mindset when they are exposed to chronic stressors. Therefore, it appears to make sense that descendants of mentally ill parents - as they are exposed to more psychosocial stressors than people with mentally healthy parents - also view stress as rather negatively, i.e. rather have a stress-is-debilitating mindset. Possibly the same problem as argued for the moderation with resilience as a moderator arose. Maybe participants with a stress-is-enhancing mindset evaluated their stressors in their childhood and adolescence retrospectively less irksome, which might have distorted results. Another possible explanation for the non-significant moderating effects of resilience and stress mindset is that the constructs examined may have been either too broad or too narrow to capture the mechanisms through which protection against stress operates in children of parents with mental illness. Research on resilience in this target group suggests that resilience is not a unitary trait, but rather emerges from a combination of specific protective processes, including access to information, social support, family functioning, connectedness, adaptive coping, and parenting factors [66]. In this regard, the present study may have assessed resilience at a comparatively broad and distal level, whereas the actual buffering processes may be more differentiated and context dependent. At the same time, stress mindset may have represented an overly narrow operationalization of stress-related coping processes. Previous research has identified efficient stress management strategies as protective for well-being in children of depressed parents [67], suggesting that stress mindset may constitute only one component influencing how stress is regulated and managed. Consequently, broader stress-management capacities rather than beliefs about stress per se may be more relevant moderators in the relationship between stressors and well-being in this population. However, previous research demonstrates the effects of the stress mindset on adaptive stress coping (e.g., [17]).

Taken together, future research should retest for the attenuating effects of the stress-mindset and resilience on the relationship between the sample-specific stressors and well-being of descendants of mentally ill parents. In doing so, future studies should use a mediating approach, preferably with a larger sample size of descendants of mentally ill parents and maybe only with those, whose parents do have an official mental health diagnosis. In order to avoid the problem with the retrospective evaluation of sample-specific stressors as described above, future studies should assess the existence of the different sample-specific stressors in the childhood and adolescence of the descendants of mentally ill parents directly (maybe even through trained professionals) and use a prospective cohort study design.

Building on looking for susceptible factors which might benefit the well-being of descendants of mentally ill parents, we checked if social identification with the family related to their well-being. Our data on this is somewhat inconsistent. Using the WHO as a well-being indicator, we found that more identification with the family related to higher well-being in descendants of mentally ill parents, whereas this could not be found using the GHQ as a well-being indicator. This might be the case because we could only find a significant difference in well-being between the two study groups for the WHO. The differing findings depending on whether well-being was assessed using the WHO or the GHQ may be explained by conceptual and methodological differences between the two instruments. While the WHO primarily captures positive aspects of subjective well-being, such as positive mood, vitality, and general life satisfaction, the GHQ is more strongly focused on psychological distress, impaired functioning, and symptoms of mental strain. Consequently, the two measures may reflect related but distinct dimensions of mental health rather than interchangeable indicators of well-being. This distinction aligns with theoretical perspectives suggesting that positive well-being and psychological distress represent partially independent constructs rather than opposite ends of a single continuum. In the context of descendants of mentally ill parents, protective factors may therefore relate differently to positive functioning versus the reduction of distress, which could explain why the observed effects varied depending on the operationalization of well-being. Assuming that the effect that more identification with the family relates to higher well-being in descendants of mentally ill parents, as indicated by the calculation using the WHO, is correct, this appears to align with previous findings. For example, Wiegand-Grefe and colleagues [68] state that reduced family functioning in families with a parental mental illness is associated with more conflicts, less adaptability and cohesion. This might probably relate to a lower social identification. The authors show, that in families with good family functioning however, children had less psychological problems compared to families with poor functioning. Children outside the clinical range of mental health symptoms where descendant from families with good family functioning and vice versa. Therefore, it can be derived that not only family functioning, but also social identification with the family can benefit the well-being of descendants of mentally ill parents. Interestingly, exploratory analyses show that descendants of mentally ill parents identify less with their family compared to people with mentally healthy parents. Cooklin [69] argues that children of mentally ill parents highly identify with that parent, yet apparently this has never been contrasted with a control group of people with mentally healthy parents.

However, as theoretically argued, identifying with a problematic group – which a family with a mentally ill parent might be – can have negative impacts as well (called “social curse” [38–40]. We therefore took a closer look at the link between social identification with the family and well-being. This led to results which contradict our previous hypotheses, but nevertheless provide interesting insights. We hypothesized, that some extent of ambivalent identification and / or disidentification might moderate the relationship between social identification with the family and well-being for descendants of mentally ill parents, in a sense that well-being would be better if ambivalent identification or disidentification were high. Contrary to our belief, we found the opposite: Whereas there was no moderating effect for disidentification, ambivalent identification moderated the relationship between identification with the family and well-being in terms of identification with the family being associated with a higher well-being when ambivalent identification is low. This effect could only be found for the descendants of mentally ill parents, not for the control group. That social identification especially manifests its positive outcomes when ambivalent identification is low has been proven in different contexts already and appears to also relate to the well-being of descendants of mentally ill parents. Ciampa and colleagues [70] depicted, that employees who had high organizational identification showed lower levels of counterproductive work behaviors, but only when ambivalent identification was low. Schuh and colleagues found a similar interaction effect: Organizational identification influenced employee’s willingness to engage in organizational citizenship behavior, but this effect was significantly reduced when employees experienced ambivalent identification [71]. This pattern is consistent with findings by Ciampa and colleagues [72], who showed that the protective effects of organizational identification on strain were stronger when ambivalent identification was low rather than high.

Thus, even though the results do not support our original hypothesis, we found that social identification with the family is rather a social cure than a social curse for descendants of mentally ill parents, despite the differences which the parental illness brings, and that it is therefore healthier for those descendants to identify with their families and have low levels of ambivalent identification. This finding has important implications for preventive mental health programs for descendants of mentally ill parents, especially as they show higher ambivalent identification (and disidentification) with their family compared to people without a mentally ill parent.

### Limitations and future research implications

One limitation of the present study is its cross-sectional design, which resulted from high dropout rates at the second measurement point and substantially limits causal interpretations of the findings. Consequently, conclusions regarding the directionality and temporal stability of the observed relationships should be drawn with caution. Nevertheless, as this study was intended as an initial step toward integrating stress mindset and social identity approaches into research on descendants of mentally ill parents, it provides a first exploratory overview of the potential relevance of these variables for this target group. Future research should therefore retest and expand the present hypotheses using longitudinal designs.

A further methodological limitation concerns the assessment of parental mental illness. Participants were included based on self-reports regarding their parents’ mental health status, and no independent clinical verification of diagnoses was conducted. Although participants were asked whether their parents had received a formal diagnosis, this information was likewise based solely on self-report. We chose this approach to facilitate recruitment and achieve the sample size indicated by the power analysis, as the study aimed to provide an initial investigation of the relevance of stress mindset and social identification in this population. However, this procedure may have increased the risk of misclassification and reduced diagnostic precision. Future studies should therefore attempt to replicate the findings using clinically verified samples, for example by recruiting participants through mental health clinics or outpatient psychotherapy services.

Another limitation is the reliance on partially retrospective self-report measures, which may be affected by recall bias, shared method variance, and subjective reinterpretations of past experiences. At the same time, constructs such as stressors, stress mindset, and social identification are inherently subjective and are therefore commonly assessed via self-report instruments. Furthermore, extending such assessments to younger children may be methodologically challenging, as these constructs require cognitive and reflective abilities that may not yet be fully developed and because the employed scales have not been validated for minors.

Future research should also examine whether the factor structure of the stressor scale can be replicated in independent samples. Although some similarities to previously proposed distinctions can be identified — for example, Cooklin [69] differentiates between fear for oneself and fear for the mentally ill parent, which resembles two of the factors identified in the present study — no established instrument currently exists that comprehensively assesses both the magnitude and the different types of stressors experienced by descendants of mentally ill parents. Consequently, the psychometric properties and generalizability of the developed scale require further validation before firm conclusions can be drawn. At least the Cronbach’s *α* = 0.91 suggests high reliability and internal consistency of the developed scale. If future studies succeed in validating this measure, it could provide important opportunities for advancing research in this field.

Finally, cultural factors were not explicitly assessed in the present study, although cultural worldviews may substantially shape the experiences of children with mentally ill parents. Beliefs about mental illness, stigma, and help-seeking differ across sociocultural contexts and may influence family communication, concealment of illness and children’s psychological adjustment [73, 74]. As these contextual influences were not controlled for in the current study, the generalizability of the findings across different sociocultural settings remains uncertain. Future research should therefore examine whether the observed effects differ across cultural contexts and explicitly include potentially relevant cultural variables and worldviews.

### Practical implications

Despite the limitations mentioned above, this study first integrates the stress mindset and social identity research into the research field of descendants of mentally ill parents and provides an overview about the differences between descendants of mentally ill parents vs. descendants of mentally healthy parents among different variables and the role which sample-specific stressors, the stress mindset, different forms of social identification and resilience play for this target group. Thus, practical implications, especially for preventative health programs for children of mentally ill parents, can be derived from our findings. First, if the newly developed assessment of sample-specific stressors is further validated, it could be used for diagnostic purposes, in order to individualize preventive health programs depending on the prevalent stressors of the children. Pretis and Dimova [62] argue that intervention programs for children of mentally ill parents should be tailored according to the specificity of the stressors the children experience. In order to do so, standardized and validated assessment tools are needed.

Furthermore, our data indicates that preventive health programs for children of mentally ill parents should include a section about social identification (and possibly the stress mindset). It appears to be beneficial for those children’s well-being to foster social identification with the family, preferably the less problematic parts of the family (e.g., if there is: with the healthy parent, siblings, positive attributes shared within the family, etc.) and simultaneously to reduce ambivalent identification. To foster social identification, parts of the Groups4Health (G4H) training [75] can be used and adjusted for this purpose. This training usually comprises five modules that are held in a group setting. The goal of this training program is to help acquire, improve and sustain social group memberships [75]. The G4H training significantly improves mental health, well-being and social connectedness [76]. Participants who complete the G4H training show improvements in depression, anxiety, stress, loneliness, and life satisfaction, which were under-pinned by participants’ increased identification with their G4H group as well as with multiple other social groups [76]. Those results are in line with our finding that a greater level of social identification is linked to a better well-being – also for descendants of mentally ill parents. Regarding ambivalent identification, it is important to note that ambivalent attitudes are more psychologically salient [77]. Costarelli and Palmonari [78] describe the affective impact of ingroup ambivalence as a result of ambivalent attitudes being more strongly influenced by negative information than by positive information about an attitude object [79] and as a result of negative information evoking stronger affective responses than positive ones [80]. Therefore, in order to reduce ambivalent identification, it might be a helpful idea to shift the attention focus from negative attributes within the family to rather positive ones, using typical techniques from cognitive-behavioral psychotherapy, e.g., cognitive reframing, mindfulness approaches to shift attention, etc. This does not mean that negative experiences regarding the parental illness or family should be ignored and invalidated, but rather that it might help those affected children to learn tools to shift their attention focus to rather positive attributes within the family, in order to not get hung up on the negative attributes, leading to a higher ambivalent identification, missing out on the positive effects of social identification with the family and thus to a worse well-being. Of course, if such elements as suggested are integrated into preventive health programs for children of mentally ill parents, their effects should be empirically evaluated.

Our data did not provide sufficient evidence for a moderating role of the stress mindset concerning the relationship between sample-specific stressors and well-being of descendants of mentally ill parents. The overall results point into the direction that this target group has a rather stress-is-debilitating mindset, which – by previous literature – is known to negatively impact well-being. Depending on future research, it might be helpful to evaluate if this target group would benefit from integrating a stress mindset training into preventive health programs. Crum and colleagues [81] developed such a training, which includes education about stress and the stress mindset and that uses cognitive reframing, in order to help participants gain a stress-is-enhancing mindset. This training has already been evaluated – although not for children and not for this target group – and significantly improves health and well-being [81].

## Conclusion

Compared to people without a parent with a mental health condition, descendants of mentally ill parents (1) viewed stress as rather negatively, thus rather had a stress-is-debilitating mindset, (2) identified less with their family and showed higher disidentification and ambivalent identification with their family and (3) reported a worse well-being (indicated by the WHO). Descendants of mentally ill parents who experienced more stressors relating to their parent’s mental health condition reported a worse well-being. Higher identification with the family related to better well-being in descendants of mentally ill parents, at least for the WHO as a well-being indicator. For this target group, disidentification did not moderate the relationship between social identification with the family and well-being. For ambivalent identification as a moderator, results show that the relationship between social identification with the family and well-being was especially high, when ambivalent identification with the family was low. This effect could only be found for the descendants of mentally ill parents, not for the control group. The relationship between the sample-specific stressors and well-being was not moderated by resilience or the stress mindset. However, in previous literature, the stress mindset and resilience show a buffering effect concerning well-being in other groups with high strain. Future research should try to replicate our findings using a longitudinal approach and control if the parents of the participants actually have a mental health diagnosis. Furthermore, the scale to assess sample-specific stressors of descendants of mentally ill parents should be validated by future research. Despite some limitations, our study integrates the stress mindset and social identity research into the research field of descendants of mentally ill parents and provides an overview about the role which sample-specific stressors, different forms of social identification, the stress mindset and resilience play for the well-being of this often overlooked group. This allows to improve much needed preventive health interventions. We propose integrating modules into preventive health programs for children of mentally ill parents, which foster social identification with the family, i.e. an adjusted version of the G4H program and reduce ambivalent identification (e.g., using cognitive reframing, mindfulness approaches to shift attention), as well as to assess the individual stressors, for example by using our sample-specific stressor list, and tailor the interventions accordingly. We also suggest testing if integrating a stress mindset training into preventive health programs provides a benefit.

## Appendix

### List of sample-specific stressors

Please rate to which extent you experienced the following stressors concerning your mentally ill parent and / or growing up with it in your childhood and adolescence.

1. Feeling of shame.
2. Feeling of guilt.
3. Fear.
4. Anger.
5. Loneliness.
6. Worries about the mentally ill parent.
7. Conflicts of loyalty (on one hand being part of the family & worrying, on the other hand feeling shame / fear / anger, etc. and wishing the situation was different).
8. Wrest of the parent due to stays at a mental health clinic.
9. Instability / Unpredictability of the mentally ill parent.
10. Taboo to speak about the parent’s mental illness in the family.
11. Taboo to speak about the parent’s mental illness outside of the family.
12. Lack of social support from outsiders.
13. Lack of understanding from outsiders.
14. Feeling of being “different” than same-aged peers; can’t identify with same-aged peers because they’re not exposed to such a situation as oneself.
15. Fear of being stigmatised oneself.
16. Negative self-perception due to the mental illness of the parent (e.g. feeling damaged, branded, anti-social, etc.)
17. Mobbing due to the mental illness of the parent or circumstances coming with it (e.g. not getting picked up by the parent from activities, lower socio-economic status, etc.)
18. Breakdown / Lack of daily structures and routines.
19. Too early takeover of the role of an adult / parent.
20. Neediness of the mentally ill parent (e.g. that you have to organize a lot for them or be emotionally available).
21. That the mentally ill parent was not available for my needs (emotional or practical needs).
22. Feeling of not being able to satisfy the duties or roles assigned to me.
23. Worrying about becoming mentally ill myself in my life.
24. Worrying about not being able to cope with life later in my life because of the stressful experiences with the mentally ill parent.
25. Breakdown of relationships in the family.
26. Constantly being in survival mode.

### Scree plot of the exploratory factor analysis

**Fig. 5:**
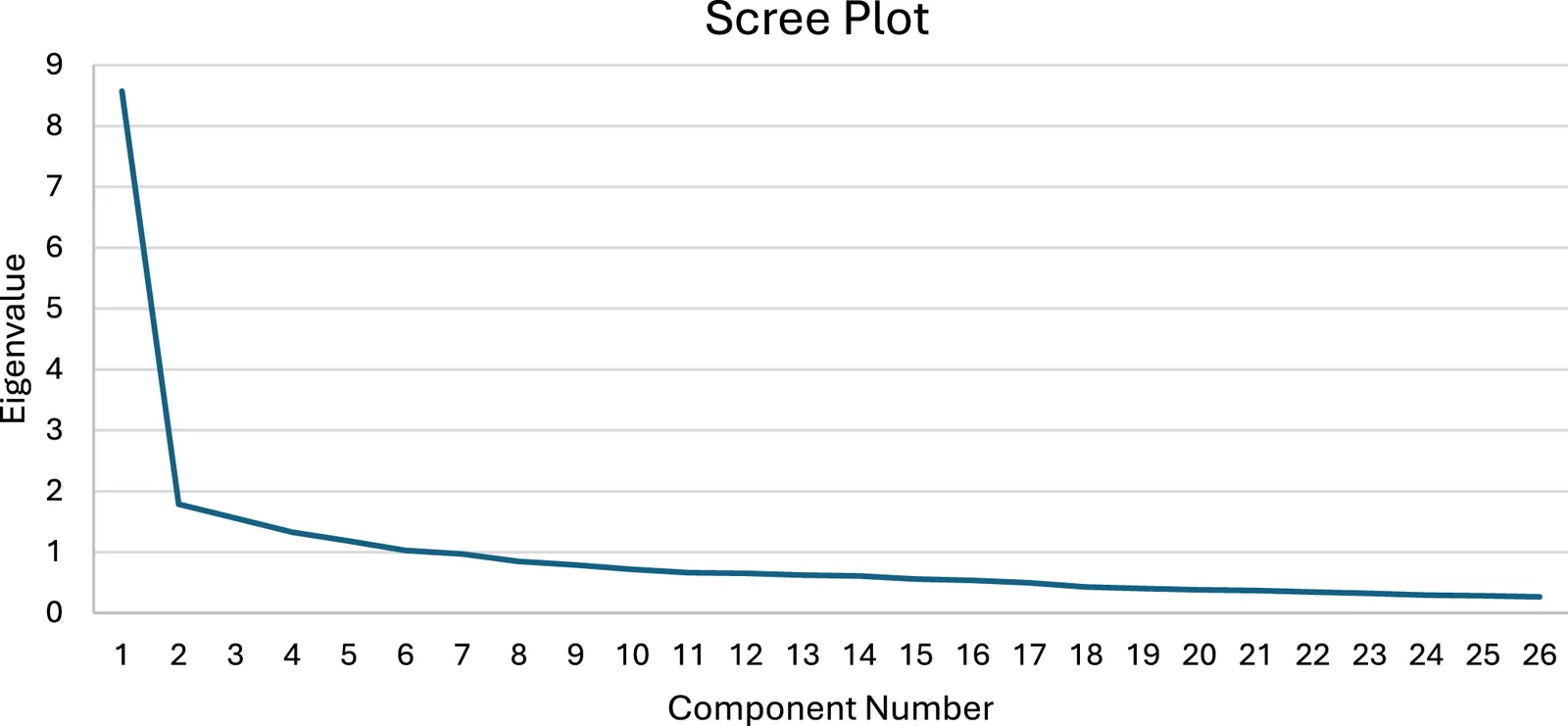
Scree plot of the factor analysis of the sample-specific stressors

## Data Availability

The dataset analyzed during the current study is available in the Open Science Framework (OSF) repository. The OSF link is available from the corresponding author upon request. The preregistration is available under: https://aspredicted.org/jk22-gpw2.pdf.

## Footnotes

1 This hypothesis was not preregistered, but as we consider it relevant on the basis of the theoretical derivation, we examine it additionally.

2 Originally, a longitudinal design with two measurement points, assessing the well-being variables a second time for both groups four weeks later was preregistered and conducted. Due to the high dropout rate (325 of the descendants of mentally ill parents; 104 of the control group) we decided to exclude the data from the second measurement point and conduct our analyses with a cross-sectional design.

3 This was preregistered differently; however, we detected an inaccuracy in the preregistration of our power analysis. We computed a new, more accurate power analysis and only report the new one here.

4 This was not preregistered.

## Abbreviations

AI: Ambivalent Identification
DI: Disidentification
DMIP: Descendants of mentally ill parents
GHQ: General Health Questionnaire
G4H: Groups4Health
Hypothesis: H (e.g. Hypothesis 1a: H1a)
IF: Family Identification
KMO: Kaiser-Meyer-Olkin Measure of Sampling Adequacy
SID Mindset: Stress-is-debilitating Mindset
SIE Mindset: Stress-is-enhancing Mindset
SMM: Stress Mindset Measure
WHO: Well-Being-Index
